# Hatching Success of Olive Ridley Sea Turtles in Semi‐Natural Nests: A Pilot Assessment of Microclimate Conditions at Batu Hiu Beach

**DOI:** 10.1002/ece3.73029

**Published:** 2026-02-03

**Authors:** Titin Herawati, Indriyani Rahayu, Ismail Maqbul, Arifikriyana Saefuramdhan

**Affiliations:** ^1^ Department of Marine Science Universitas Padjadjaran Sumedang West Java Indonesia; ^2^ Master of Marine Conservation Universitas Padjadjaran Sumedang West Java Indonesia

**Keywords:** hatching success, incubation microclimate, *Lepidochelys olivacea*

## Abstract

Olive ridley sea turtles ( *Lepidochelys olivacea* ) continue to experience population pressures across much of their global range, underscoring the need to understand how incubation environments influence reproductive outcomes in conservation settings. Semi‐natural hatcheries are widely used in Indonesia, yet quantitative assessments of their microclimatic performance remain limited. This pilot study examined the hatching success of three olive ridley clutches relocated from natural nests to a semi‐natural hatchery at Batu Hiu Beach, West Java, between January and May 2025. A total of 303 eggs were relocated within 6 h of oviposition, placed into hand‐dug nests that mimicked natural nest dimensions, and incubated under conditions designed to replicate beach microhabitats. Microclimate variables, including daily air temperature and relative humidity, were extracted from ERA5 reanalysis data and matched to each incubation period. Hatching outcomes varied considerably among the three incubation windows, with success rates ranging from 41% to 95%. The clutch incubated under cooler, more thermally stable conditions had the highest success, while the clutch exposed to more variable temperatures had the lowest success. Humidity varied slightly among periods and showed no clear correspondence with differences in reproductive output. These findings suggest the importance of fine‐scale thermal stability in supporting embryonic development, consistent with broader evidence from tropical rookeries where temperature is a key driver of reproductive success. The results provide preliminary guidance for improving hatchery management through targeted shading, moisture regulation, and timing of nest relocation. This pilot assessment across three clutches emphasizes the need for multi‐season monitoring and in‐nest data logging to refine conservation strategies for *L. olivacea* in Indonesia.

## Graphical abstract

Our study investigates the environmental and astronomical drivers of olive ridley (Lepidochelys olivacea) hatching success at Batu Hiu Beach, Indonesia. We demonstrate that air temperature is the dominant predictor of hatching outcomes, while Earth–Moon distance exerts an additional, significant influence independent of humidity and tidal effects. These findings highlight the importance of integrating climatic and astronomical factors into predictive models for sea turtle conservation under changing climate conditions.

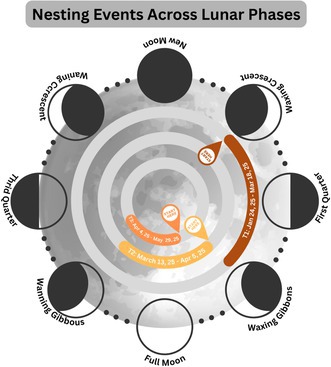

## 1 Introduction

The olive ridley sea turtle ( *L. olivacea* ) is currently listed as *Vulnerable* by the International Union for Conservation of Nature (IUCN) Red List, with global populations declining approximately 30%–50% in recent decades (Cáceres‐Farías et al. 2022; Labastida‐Estrada et al. 2024). Despite being historically considered one of the most abundant sea turtle species, escalating anthropogenic pressures have severely impacted its survival. The species' wide distribution across tropical regions of the Atlantic, Pacific, and Indian Oceans exposes it to a multitude of threats, including habitat destruction, overharvesting of eggs, and incidental capture in fishing gear that vary in intensity and form across regions (Cáceres‐Farías et al. 2022; Morales‐Mérida et al. 2022). Long‐term demographic monitoring in major arribada sites such as Odisha (India), La Escobilla (Mexico), and Ostional (Costa Rica) further documents multi‐decade declines in nesting numbers despite periodic high‐density nesting events (Pheasey et al. 2021; An et al. 2025; Negus et al. 2025).

Among the most pressing threats is bycatch, especially in artisanal and industrial fisheries where turtles often become entangled in gillnets, longlines, and trawl gear. Such interactions not only result in direct mortality but also increase risks of gas embolism and physiological trauma (Crespo‐Picazo et al. 2020; Vera et al. 2021; Chomchat et al. 2023). In addition, coastal habitat degradation caused by urbanization, tourism, and pollution has significantly altered beach morphology and nesting suitability in many regions, including Indonesia (Bahri et al. 2023). Moreover, egg poaching remains widespread in areas with weak law enforcement or strong cultural dependence on sea turtle eggs, such as in parts of East Java, Nusa Tenggara Barat, and Southeast Sulawesi (Rosalina and Prihajatno 2022; Maulana et al. 2024). Plastic pollution also poses a growing threat, with olive ridleys exhibiting documented high ingestion rates in several rookeries worldwide (López‐Martínez et al. 2020). Emerging diseases, such as *Fusarium* spp. infections that cause egg fusariosis and embryonic mortality, further complicate conservation challenges (Gleason et al. 2020; Saenz 2023).

The reproductive ecology of *L. olivacea* includes unique adaptations, such as the arribada phenomenon, a synchronous mass‐nesting event in which thousands of females lay eggs simultaneously on specific beaches (Buenrostro‐Silva et al. 2024; Faddilah et al. 2024). This behavior, observed in regions such as Costa Rica, Mexico, and the Indian subcontinent, is thought to enhance offspring survival by predator satiation and is influenced by environmental factors such as sea surface temperature, lunar cycles, and substrate quality (Gallego et al. 2020; Setyorini et al. 2025). While solitary nesting is more common in Indonesia, arribada‐like behavior has been sporadically reported in select rookeries (Setyorini et al. 2025). For successful incubation, coastal nesting habitats with soft‐grained sand, stable thermal regimes, and minimal anthropogenic disturbance are required (Brodie et al. 2020; Manurung 2023). Unfortunately, these optimal conditions are increasingly rare due to unchecked coastal development. In parallel, long‐term nesting datasets from Mexico, India, and Sri Lanka suggest that the frequency and scale of arribadas are themselves sensitive to climatic anomalies and local habitat changes, indicating ecological vulnerability (Robinson et al. 2021).

Temperature plays a pivotal role in sea turtle reproduction through temperature‐dependent sex determination (TSD), in which higher incubation temperatures result in female‐biased hatchling populations (Lockley and Eizaguirre 2021; Silva et al. 2025). This feminization trend is already evident in several populations, with some reporting > 99% female hatchlings (Booth et al. 2020; Barraza et al. 2023). Climate change amplifies this imbalance and affects incubation success, embryonic development, and nesting phenology. Projections suggest a global increase in sea temperatures by 0.58°C–4.17°C by the end of the century, potentially rendering many rookeries unsuitable for viable reproduction (Fuentes et al. 2023; Staines et al. 2023). For olive ridleys, pivotal temperatures of ~29°C–30°C have been widely cited, although localized deviations can occur due to microhabitat variation, adding uncertainty to future sex‐ratio predictions under warming scenarios (Candan 2023; Morales‐Mérida et al. 2023; Tromp et al. 2025). Compounding this issue is the long maturation period of sea turtles, which delays the population‐level consequences of current sex‐ratio skew (Heppell et al. 2022; Hatase et al. 2024). Although some populations show signs of behavioral or nesting‐site shifts that may buffer thermal stress, empirical evidence remains limited and inconsistent (Young et al. 2023; Crowther and Schwanz 2025).

In Southeast Asia, particularly Indonesia, egg harvesting has long been embedded in local socio‐economic systems. Traditional and subsistence hunting often overlap with illegal trade networks, posing a significant challenge for conservation enforcement (Sas‐Rolfes et al. 2019; Tilker et al. 2019). In some communities, such as Maluku, egg collection is part of customary practices and is not necessarily viewed as ecologically harmful (Simanjuntak et al. 2020; Leimena et al. 2023). However, increasing market demand and habitat loss have pushed many species toward local extinction thresholds. Studies emphasize the need for culturally sensitive, multi‐stakeholder conservation models that integrate ecological science with community engagement and governance reforms (Estrada et al. 2020; Jiao et al. 2021). The Ostional program in Costa Rica exemplifies such integration, where legalized egg harvest has reduced poaching, strengthened community stewardship, and improved socioeconomic conditions while maintaining stable nesting numbers (Bézy et al. 2020; Jani et al. 2020; Pheasey et al. 2023).

In response to these threats, semi‐natural hatcheries have emerged as a promising conservation tool. These systems mimic natural nesting conditions while protecting from predators, extreme weather, and human disturbance. Empirical studies on sea turtles and other taxa, such as penguins, cranes, and seabirds, have demonstrated that artificial or semi‐natural nesting strategies can enhance hatching success and reproductive output (Edwards et al. 2021; Burke et al. 2022; Pichegru et al. 2024). In Indonesia, initiatives in Pangumbahan and Lombok have shown positive results using this approach (Fitri et al. 2022). Nevertheless, maintaining optimal thermal and humidity conditions remains challenging, particularly under fluctuating climatic conditions. Recent performance reviews from Indonesian hatcheries report highly variable hatching success depending on sand temperature, rainfall, moisture inputs, and nest depth, reaffirming the importance of microclimate regulation (Sukandar et al. 2020; Faddilah et al. 2024).

Despite the growing number of studies on the role of temperature and humidity in sea turtle reproductive success, few have investigated the combined influence of environmental and astronomical variables, such as lunar phases, tidal patterns, and especially Earth–Moon distance, on the nesting behavior and hatching outcomes of olive ridley turtles, especially in Southeast Asian conservation contexts. Environmental factors, including ENSO‐linked warming, rainfall pulses, sand moisture, flooding, and solar radiation, have been documented to influence incubation success, yet remain underexplored in the Indonesian context (Tomillo et al. 2020; Matthews et al. 2021; Carpio et al. 2022; Wiggins et al. 2023). This lack of integrated ecological‐astronomical analysis constitutes a critical research gap in the design of adaptive conservation strategies.

This study aims to analyze the hatching success rate of *L. olivacea* eggs in semi‐natural nests at Batu Hiu Beach, Pangandaran, Indonesia, and to investigate the effects of environmental variables, including air temperature, humidity, lunar phases, tidal elevations, and Earth–Moon distance, on hatching outcomes. The novelty of this research lies in moving beyond temperature‐focused models by explicitly incorporating lunar distance into predictive frameworks of hatching success. By integrating climatic and astronomical factors, this study provides actionable insights for site‐specific, evidence‐based conservation of olive ridley turtle populations in Indonesia.

## 2 Materials and Methods

### 2.1 Study Area and Sampling Design

**FIGURE 1:**
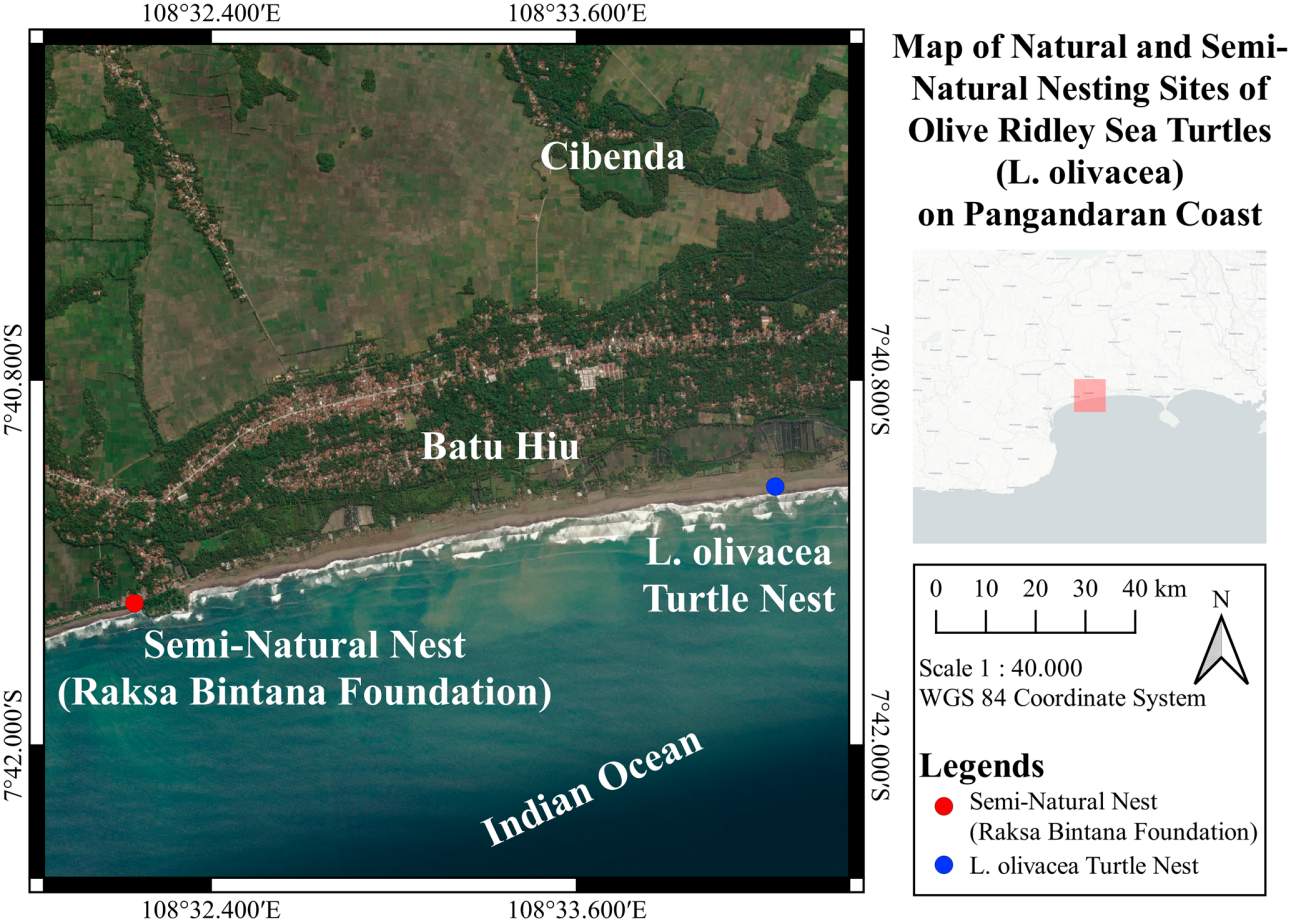
Study area and sampling design.

This study was conducted from January to May 2025 in the coastal area of Pangandaran Regency, southern Java, Indonesia (Figure 1). Natural nesting activity was recorded in Cibenda Village (7°41′7.17″ S, 108°34′22.06″ E), and all retrieved eggs were relocated to the Raksa Bintana Foundation hatchery at Batu Hiu Beach (7°41′32" S, 108°32′9" E), approximately 3 km from the nesting site. The hatchery provides a semi‐natural incubation environment that resembles natural beach conditions while protecting against disturbance and predators.

Three nesting events occurred during the study period. The first clutch (80 eggs) was collected on 24 January 2025, the second clutch (135 eggs) on 13 March, and the third clutch (88 eggs) on 8 April. In all cases, eggs were relocated within 6 h of oviposition, as females nested during the night and eggs were transferred the following morning. During relocation, eggs were placed in buckets or plastic bags lined with sand to minimize vibration. Egg orientation was maintained and not rotated, in accordance with standard sea turtle handling procedures to prevent embryo detachment.

For the second clutch, all 135 eggs represented the entire clutch, and none were left behind or damaged at the nesting site. All eggs were buried in hand‐dug nest chambers at the hatchery, approximately 40–50 cm deep, mimicking the dimensions and shape of natural nests. Nest chambers were covered with sand and shaded using mesh and cloth to reduce exposure to direct sunlight and rainfall.

### 2.2 Environmental and Astronomical Variables

#### 2.2.1 Tidal Data

Several abiotic and astronomical variables were monitored throughout the study to evaluate the influence of environmental factors on hatching outcomes. Tidal elevation data were obtained from the Geospatial Information Agency of Indonesia (BIG) through the official portal (https://srgi.big.go.id), using hourly predictions based on geodetic tide models. The monitoring point was near the hatchery site (–7.6926° S, 108.5359° E). From this dataset, daily maximum and minimum tide values were extra.cted and matched with nesting and incubation dates.

#### 2.2.2 Lunar Phases and Earth–Moon Distance

Lunar phase data were compiled from the NASA Moon Observation Guide and Timeanddate.com, while the Earth–Moon distance was retrieved from the Indonesian Meteorological, Climatological, and Geophysical Agency (BMKG). These variables assessed whether astronomical factors, such as moonlight intensity or lunar proximity, influenced nesting activity or incubation success.

#### 2.2.3 Air Temperature and Humidity

Air temperature and humidity were calculated using reanalysis data from the ERA5 dataset provided by the European Centre for Medium‐Range Weather Forecasts (ECMWF), accessed through the Copernicus Climate Data Store. Variables included 2 m air temperature and dew point temperature, extracted at 6 h intervals and averaged daily. Temperature values were converted from Kelvin to Celsius. Relative humidity was calculated using the Magnus equation, which estimates vapor pressure at air and dew point temperatures. All processing was carried out in Microsoft Excel, with verification scripts written in Python to check for data consistency.

#### 2.2.4 Hatching Success and Incubation Period

Hatching success (%) was calculated as the number of viable hatchlings divided by the total eggs per clutch. Each egg was categorized as hatched, sterile, or failed to hatch. In this study, sterile eggs were defined as unfertilized eggs that showed no embryonic development. Morphologically, sterile eggs contained only liquid or gelatinous yolk, no visible embryonic tissue, and no blood vessels or vascularization. Failed‐to‐hatch eggs were classified into three categories based on morphological criteria commonly used in sea turtle embryology: Early‐stage mortality (embryo not visible; presence of intact yolk or a blood ring; pale yolk coloration; development ceased before organogenesis), mid‐stage mortality (partial embryo visible (e.g., head or limb buds); reduced or degraded vascularization; tissue darkening or necrosis; incomplete organ formation), late‐stage mortality (dead‐in‐shell) (fully formed or nearly formed hatchling that failed to emerge; tissues may appear darkened, shrunken, or desiccated; no active vascularization). Incubation period was defined as the number of days between burial and the first emergence of hatchlings.

### 2.3 Descriptive Data Analysis

Given the small number of clutches, analyses focused on descriptive patterns rather than inferential statistics. Hatching success, incubation duration, temperature, humidity, tidal amplitude, and Earth–Moon distance were summarized using means, standard deviations, and ranges. Differences among clutches were interpreted qualitatively to identify potential environmental associations.

### 2.4 Data Visualization Strategy

To aid interpretation and communication of results, a comprehensive data visualization strategy was implemented using the ggplot2, ggpubr, and ggalluvial packages in R. Boxplots were generated to compare temperature and humidity distributions across treatment groups. At the same time, bar charts with standard error bars illustrated hatch success and its variation. Scatterplots were used to visualize the regression relationships between hatching success and environmental variables. Timeline charts were developed to present the duration and overlap of the three incubation periods on a calendar scale. Additionally, a Sankey diagram was used to display the flow of egg outcomes from initial collection to final fate (hatched, sterile, failed), and moon‐phase icons were overlaid on nesting‐date timelines to explore potential lunar associations visually. All figures were exported in high‐resolution formats suitable for scientific publication.

## 3 Results and Discussion

### 3.1 Results

#### 3.1.1 Nesting Events and Environmental Conditions

During the 2025 nesting season at Batu Hiu Beach, three olive ridley turtles ( *Lepidochelys olivacea* ) deposited eggs on separate dates. These events occurred on 24 January (T1), 13 March (T2), and 8 April (T3). Each nesting event took place under different lunar and tidal conditions (Table 1).

**TABLE 1:** Environmental conditions during *L. olivacea* nesting events at Batu Hiu Beach, 2025.

Treatment	Nesting Date	Moon Phase	Tidal Amplitude (m)	Earth–Moon Distance (km)
T1	24 Jan 2025	Waning Crescent	0.707	397,256
T2	13 Mar 2025	Waxing Gibbous	1.285	399,714
T3	08 Apr 2025	Waxing Gibbous	0.710	396,853

T1 occurred during a waning crescent moon with a tidal amplitude of 0.707 m and an Earth–Moon distance of 397,256 km. T2 took place during a waxing gibbous phase, coinciding with the highest tidal amplitude recorded in this study (1.285 m) and the most significant Earth–Moon distance (399,714 km). T3 also occurred during a waxing gibbous phase, although with a much lower tidal amplitude (0.710 m) and the closest Earth–Moon distance (396,853 km).

**FIGURE 2:**
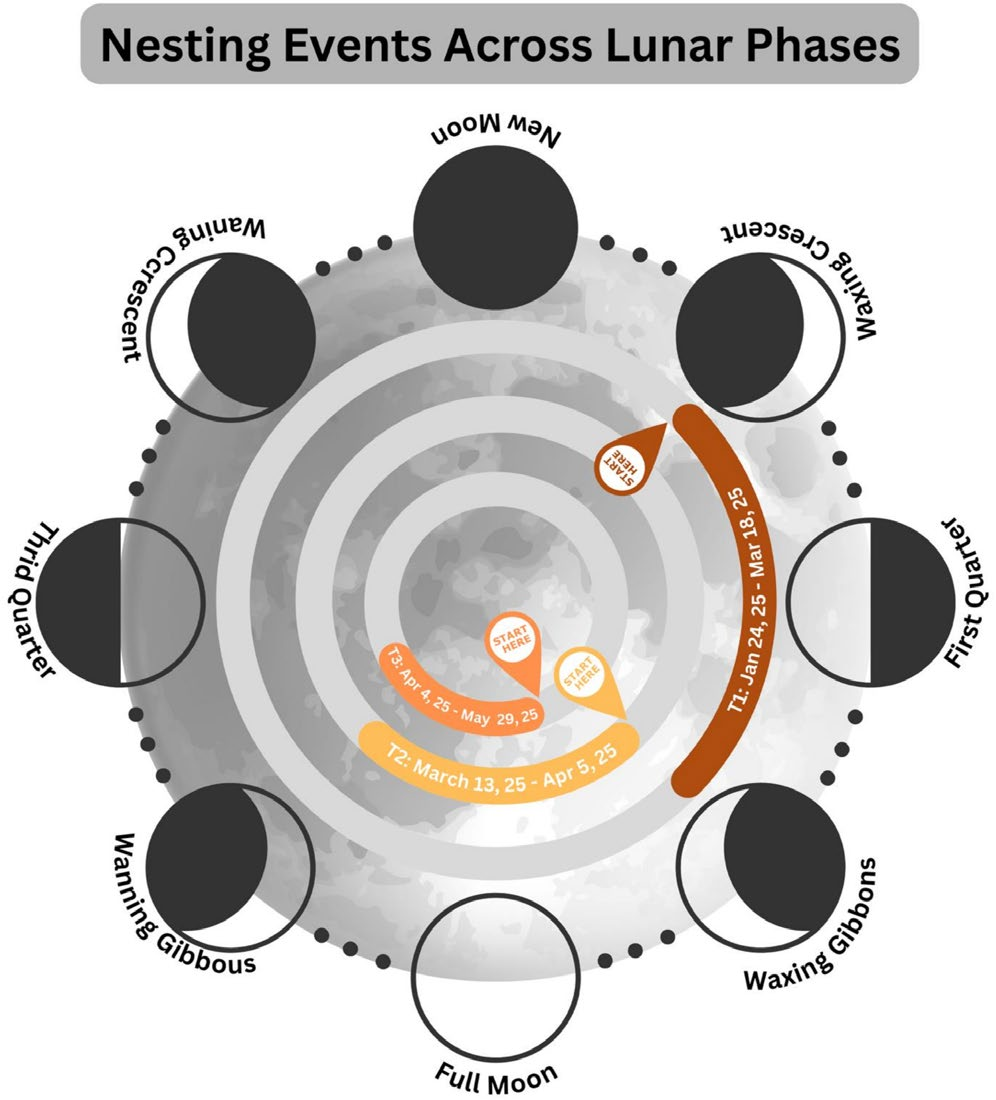
Nesting events of *L. olivacea* mapped across the lunar cycle. Two events occurred during the waxing gibbous phases (T2 and T3), while one occurred during a waning crescent (T1), indicating no consistent lunar phase synchronization.

**FIGURE 3:**
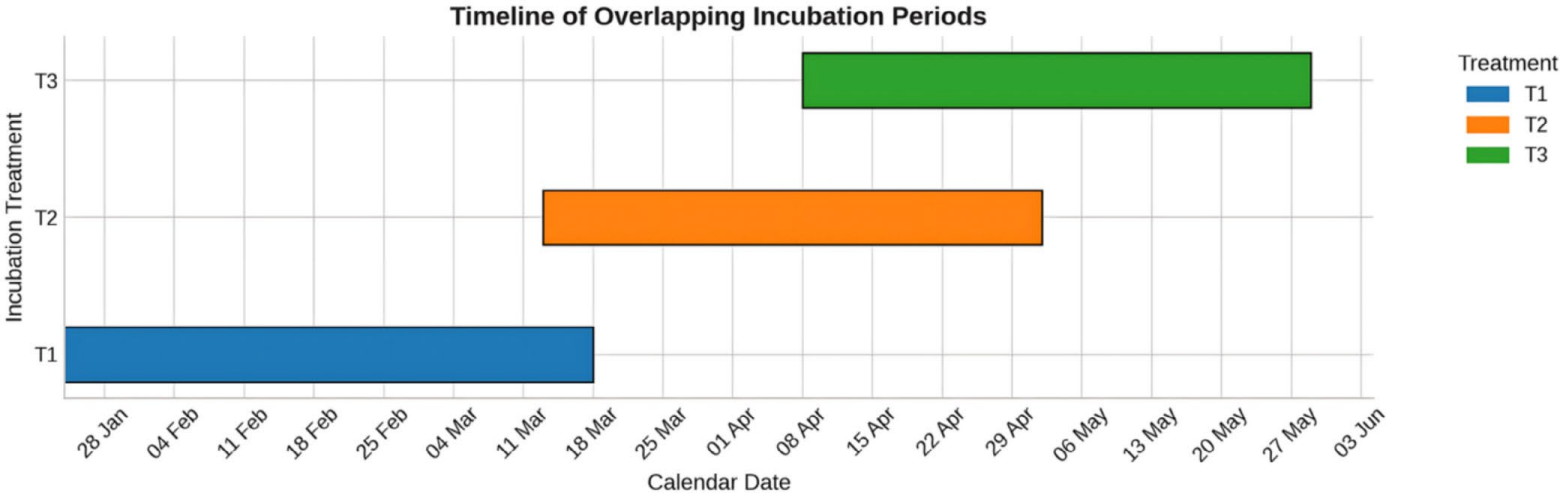
Timeline of overlapping incubation periods across the three treatments. Incubation durations ranged from 51 to 53 days, showing temporal overlap particularly between T2 and T3.

These observations illustrate that nesting activity occurred across different lunar phases and tidal conditions. No consistent pattern linking nesting events to specific lunar or tidal states can be identified from this small set of observations. The three nesting dates and the corresponding incubation ranges are shown in Figures 2 and 3.

#### 3.1.2 Incubation and Hatching Output

A total of 303 eggs from three *L. olivacea* clutches were monitored across the three incubation treatments (T1, T2, and T3), corresponding to nesting events between January and April 2025. Incubation periods ranged from 51 to 53 days and showed partial temporal overlap (Figure 3). T1 incubated from 24 January to 18 March (53 days), T2 from 13 March to 2 May (51 days), and T3 from 8 April to 29 May (52 days). This overlap reflects the seasonal sequence of nesting and indicates that each clutch experienced a slightly different environmental context.

Hatching output varied among the three clutches (Table 2). T1 produced the highest hatching success (95.0%; 76 hatchlings from 80 eggs), with a small proportion of sterile eggs (5.0%) and no recorded developmental failures. T2 showed the lowest success (44.4%; 60 hatchlings from 135 eggs), along with the highest proportion of sterile eggs (29.6%) and developmental failures (25.9%). T3 yielded an intermediate hatching success (60.2%; 53 hatchlings from 88 eggs), with 32.9% sterile eggs and 6.8% developmental failures.

**TABLE 2:** Hatching outcomes and incubation duration for each treatment group.

Treatment	Total Eggs	Hatchlings *n* (%)	Sterile *n* (%)	Failed *n* (%)	Incubation Period
T1	80	76 (95.0%)	4 (5.0%)	0 (0.0%)	24 Jan–18 Mar (53 days)
T2	135	60 (44.4%)	40 (29.6%)	35 (25.9%)	13 Mar–2 May (51 days)
T3	88	53 (60.2%)	29 (32.9%)	6 (6.8%)	8 Apr–29 May (52 days)

**FIGURE 4:**
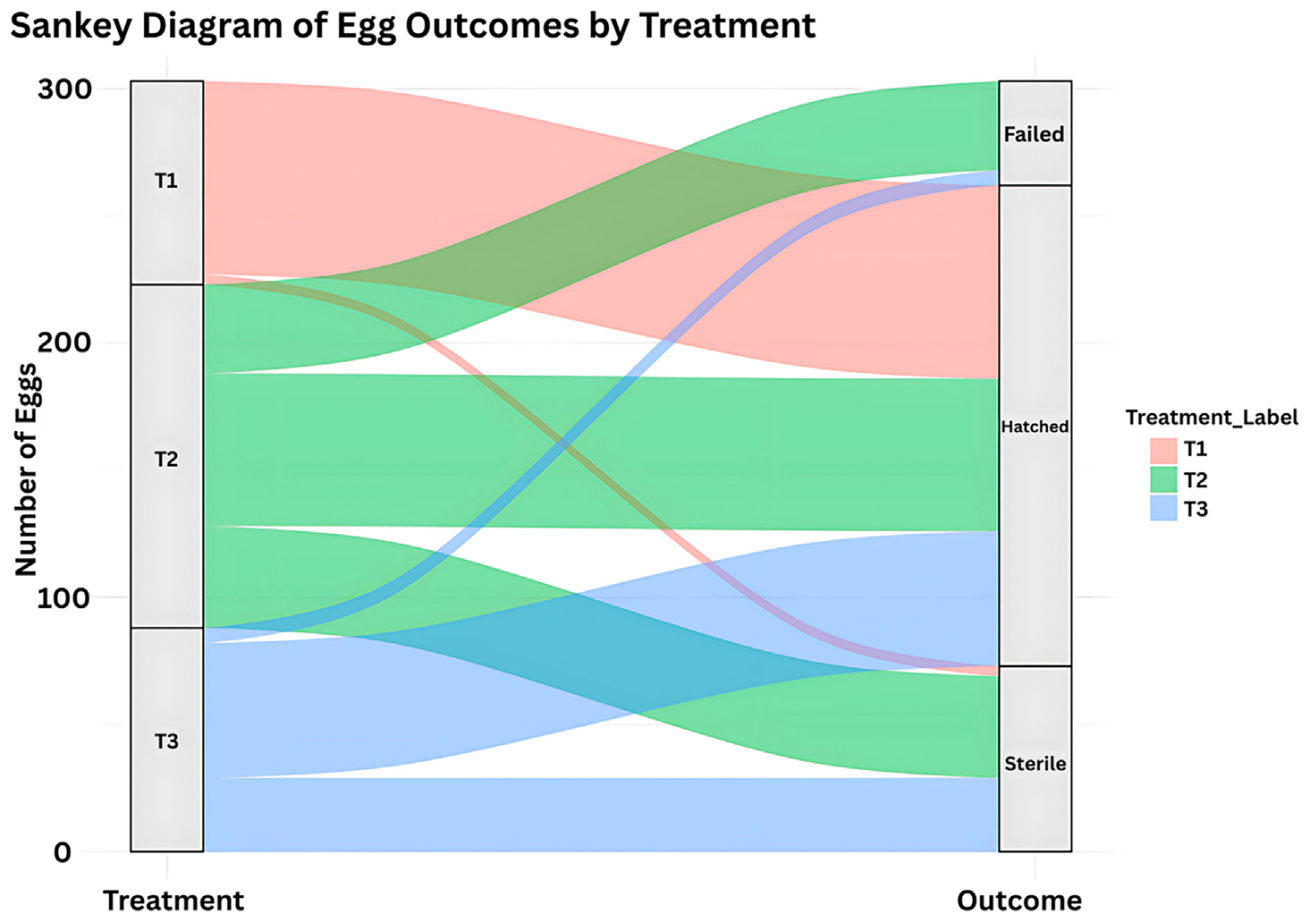
Sankey diagram of egg outcomes for *L. olivacea* across three incubation treatments. The flow diagram illustrates the fate of 303 eggs monitored in three incubation treatments: T1, T2, and T3. The width of each stream corresponds to the number of eggs, resulting in three outcome categories: Hatched, sterile, or failed to hatch. Treatment 1 yielded the highest proportion of hatchlings (red), while Treatment 2 (green) exhibited the lowest hatching success and the highest rates of sterility and embryonic failure. Treatment 3 (blue) demonstrated moderate outcomes across all categories.

These outcomes are illustrated in the Sankey diagram (Figure 4), which displays the distribution of eggs across the categories of hatched, sterile, and developmentally failed. The diagram highlights the substantial variation among clutches, reflecting differences in overall reproductive output. As this study is based on only three nests, these patterns should be interpreted cautiously, but they provide valuable baseline information for understanding clutch‐level variability at this site.

#### 3.1.3 Physical Parameters (Temperature and Humidity)

Daily ambient air temperature and relative humidity during each incubation period were obtained from the ERA5 reanalysis dataset. These measurements provide a general description of the broader environmental conditions experienced during incubation, although they do not directly represent temperatures or humidity within the nest.

Mean temperature and humidity differed slightly among the three incubation periods (Table 3). T1 had a relatively stable temperature profile (26.67°C ± 0.52°C) and moderate humidity (83.93% ± 2.88%). T2 showed the widest temperature variation (25.74°C ± 1.15°C) and similar mean humidity to T1 (84.11% ± 2.76%). T3 exhibited intermediate temperatures (26.48°C ± 0.51°C) and the highest mean relative humidity (87.01% ± 2.92%).

**TABLE 3:** Summary of mean daily temperature and relative humidity during the incubation period for each treatment.

Treatment	Incubation Period	Mean Temp (°C)	SD Temp	Mean RH (%)	SD RH
T1	24 Jan–18 Mar (53 days)	26.67	0.52	83.93	2.88
T2	13 Mar–2 May (51 days)	25.74	1.15	84.11	2.76
T3	8 Apr–29 May (52 days)	26.48	0.51	87.01	2.92

**FIGURE 5:**
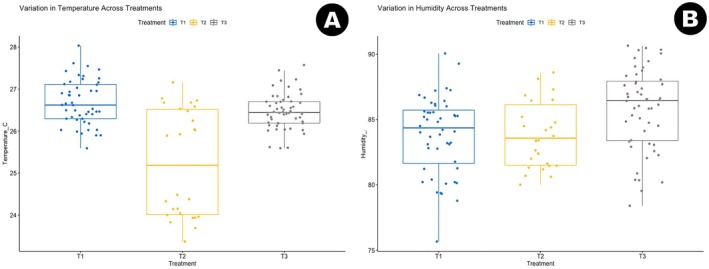
Variation in incubation microclimate across treatment groups. (A) Boxplot of daily average air temperature (°C) across treatments T1 (blue), T2 (yellow), and T3 (gray). (B) Boxplot of daily relative humidity (%) across the same treatment groups. T1 exhibited the lowest temperature variability and highest humidity, while T2 showed wider variation, and T3 was associated with the warmest and driest conditions.

These patterns are shown in Figure 5, which displays boxplots of temperature and humidity for each treatment group. Although temperature and humidity varied among incubation periods, the small number of nests in this study limits interpretation. The observed environmental profiles provide descriptive context for each clutch but do not allow conclusions about causal relationships with hatching success.

#### 3.1.4 Hatching Success Variation

Hatching success differed among the three clutches, with T1 showing the highest value (95.0%), followed by T3 (60.2%) and T2 (44.4%) (Table 2). These differences illustrate substantial clutch‐level variability in reproductive output. Because only three nests were available, we did not conduct inferential statistical tests comparing treatments. Using daily environmental measurements as independent replicates would artificially inflate sample size and lead to pseudoreplication, as noted by previous studies and reviewers. For this reason, the analysis is limited to descriptive comparisons.

**FIGURE 6:**
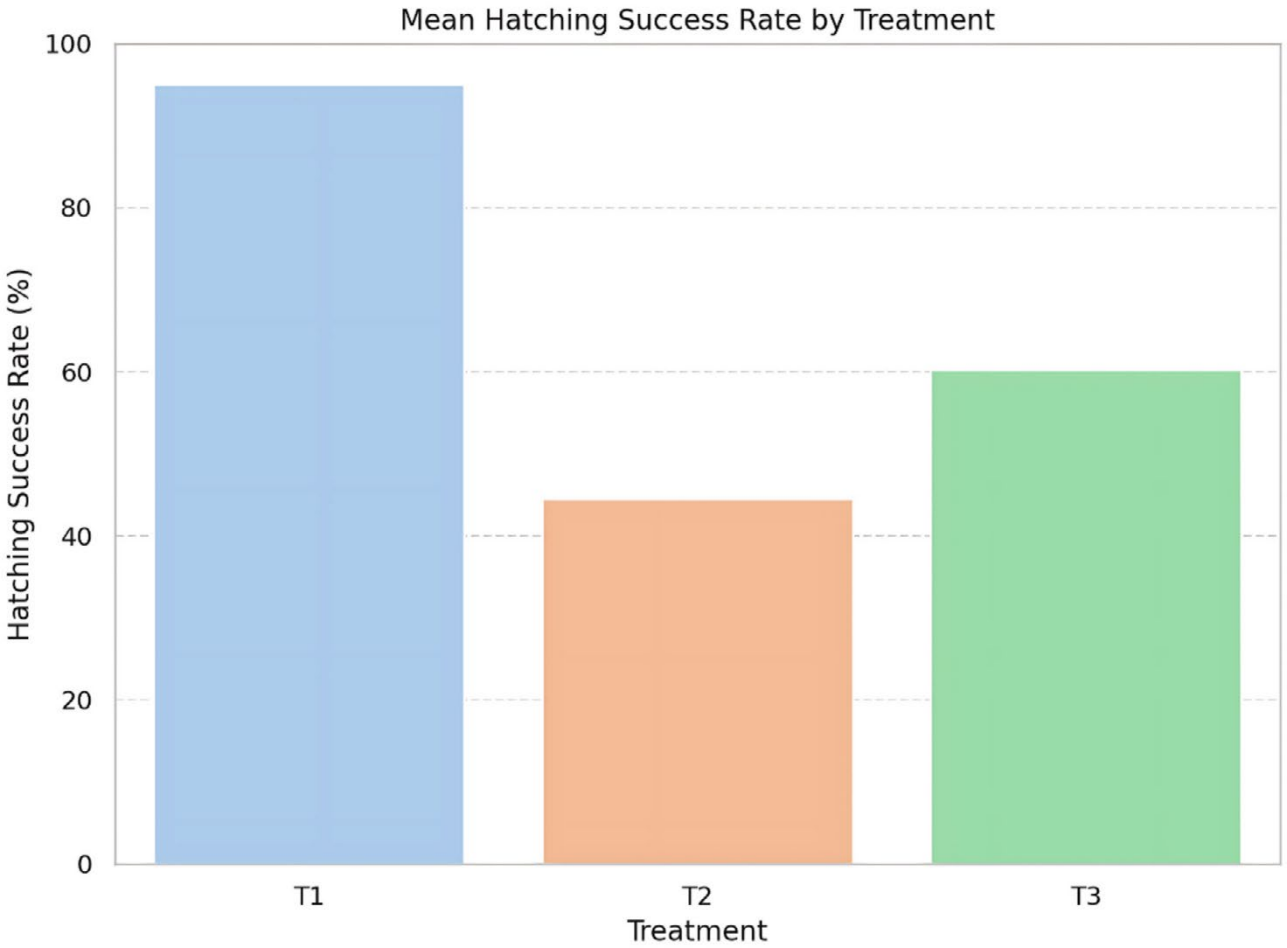
Mean hatching success rate (%) across incubation treatments. Bar plot showing the average hatching success rate for each treatment. T1 (light blue) had the highest success rate (~95%), followed by T3 (green, ~60%), and T2 (orange, ~45%). These differences are consistent with post hoc statistical results (Tukey HSD, *p* < 0.001).

A visual summary of hatching success across the three clutches is provided in Figure 6. T1 exhibits the highest proportion of successfully hatched eggs, whereas T2 shows the lowest. T3 displays intermediate outcomes. These patterns reflect variation among individual nests and provide a baseline for hatchery performance at this site.

Although environmental conditions differed among incubation periods (Table 3), the small number of clutches prevents assessment of statistical relationships between microclimate and hatch outcomes. Instead, the descriptive trends may help guide hypotheses for future work with larger datasets, particularly regarding temperature stability and moisture conditions. However, no causal relationships can be inferred from the present study.

#### 3.1.5 Exploratory Assessment of Environmental Factors

Environmental factors were examined descriptively at the clutch level, and exploratory plots were used to visualize potential patterns. These observations should be viewed as preliminary, given the small number of nests.

Visual inspection of clutch‐level averages suggests that T1, which had the highest hatching success, also experienced relatively stable temperatures and moderate humidity during incubation (Table 3). T2, with the lowest hatching success, showed the widest temperature variation. T3 exhibited high relative humidity and intermediate hatching output. These patterns, however, remain preliminary and should not be interpreted as evidence of causal relationships.

**FIGURE 7:**
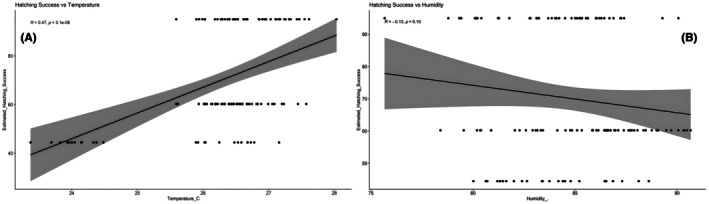
Relationships between environmental parameters and hatching success in *L. olivacea* . (A) A significant positive linear relationship is observed between air temperature (°C) and hatching success (%), indicating that increased incubation temperatures enhance embryonic development and emergence success (*p* < 0.001). (B) No significant association was found between relative humidity (%) and hatching success (*p* = 0.151), suggesting minimal influence of humidity within the observed range.

Plots of clutch‐level temperature, humidity, and hatching success (Figure 7) provide a descriptive view of potential environmental trends. For example, higher mean temperatures appeared to be associated with higher hatching success across the three clutches; however, this trend cannot be statistically evaluated with the present dataset. Similarly, although lunar and tidal conditions varied across nesting dates, no conclusions can be drawn about their influence on hatching outcomes.

Overall, these exploratory observations highlight patterns that may be useful in designing future studies with larger sample sizes or multi‐season monitoring. The present results should therefore be interpreted as hypothesis‐generating rather than hypothesis‐testing.

### 3.2 Discussion

#### 3.2.1 Interpretation of Hatching Success Variation Across Treatments

The three monitored clutches exhibited marked differences in hatching success, with the clutch incubated under the coolest and most thermally stable conditions achieving the highest success. In contrast, the other two clutches showed reduced outcomes. This pattern aligns with broader observations from tropical rookeries such as Binasi Beach (Manurung 2023) and Ostional Beach, Costa Rica (Valverde et al. 2010), where nests experiencing moderate and stable temperatures typically yield higher viability.

Temperature thresholds documented in multiple studies, where success declines above 31°C and prolonged exposure beyond 35°C increases embryonic mortality and developmental abnormalities (Morales‐Mérida et al. 2022; Sandoval‐Ramírez and Solana‐Arellano 2023), plausibly explain the differences observed among treatments. These thermal sensitivities are consistent with reports from Central American *L. olivacea* populations, where elevated incubation temperatures not only reduce success but also skew sex ratios due to temperature‐dependent sex determination (Abreu‐Grobois et al. 2020; Sosa‐Cornejo et al. 2021).

Equally important is thermal stability. Microhabitats with narrow daily temperature ranges reduce developmental stress, enhance gas exchange, and prevent desiccation (Gleason et al. 2020; Taylor et al. 2020). The high‐performing clutch in this study experienced the narrowest thermal range, echoing findings that shaded or vegetated nesting sites reduce overheating and promote balanced sex ratios (Saura et al. 2022; Sullivan et al. 2022). Conversely, broader fluctuations, as observed in the lowest‐performing clutch, are associated with increased risk of embryonic mortality (Adams et al. 2022; Veelenturf et al. 2022).

Humidity varied moderately among treatments, and although there was no apparent independent effect, previous work highlights its importance in preventing desiccation and maintaining egg hydration (Bomfim et al. 2021; Zhang et al. 2025). Extreme deviations in moisture disrupt oxygen diffusion and embryonic water balance, with interactions between humidity and temperature shaping viability and hatchling morphology (Fuentes et al. 2023; Trewartha et al. 2024). The relatively stable moisture levels in the highest‐performing clutch may have contributed to a more favorable developmental environment.

Differences in incubation duration also corresponded with microclimatic variation. Favorable thermal conditions allowed the most extended developmental period (~53 days), while more variable regimes may have disrupted critical developmental phases (Carpio et al. 2022). Global comparisons similarly indicate that suitable thermal conditions, moderate humidity, and minimal variation in temperature and humidity support higher hatch rates (Laloë and Hays 2023; Ceolotto et al. 2025).

### 3.3 Role of Temperature and Humidity as Environmental Drivers

The descriptive patterns observed in this study suggest that temperature is a key factor associated with hatching success in *L. olivacea* , in line with optimal incubation temperatures of 28°C–32°C (Morales‐Mérida et al. 2023; Labastida‐Estrada et al. 2024). Fluctuations above 35°C can accelerate embryogenesis, shortening incubation length (Matthews et al. 2021; Dong et al. 2022), but at the cost of hatchling performance (Fleming et al. 2020). These supra‐optimal peaks have been linked to reduced locomotor capacity, compromised dispersal ability, and lower post‐emergence survival (Morales‐Mérida et al. 2021; Dong et al. 2022).

Humidity, while not strongly variable across treatments here, has well‐established roles in sea turtle incubation. Relative humidity above 69% has been associated with improved success in *Eretmochelys imbricata* (Bomfim et al. 2021). Low moisture contributes to desiccation and reduced emergence (Flores‐Aguirre et al. 2023), whereas excessive moisture or flooding, especially during late incubation, can cause hypoxia and pathogen exposure (Limpus et al. 2021; Carpio et al. 2022). The lack of an apparent humidity effect in this dataset may reflect a narrow environmental range and the substrate's buffering capacity (Matthews et al. 2021).

The complexity of temperature–humidity interactions has been noted in various studies (Martín‐del‐Campo et al. 2021; Wei et al. 2021). Extreme conditions in one factor often magnify stress from the other. In this context, the observed values remained within tolerable boundaries for *L. olivacea* , helping explain why humidity did not independently correspond with variation in outcomes.

### 3.4 Possible Lunar–Tidal Influences on Microclimate

Although not a primary objective, the timing of clutch deposition overlapped with varying stages of the Earth–Moon distance cycle. The clutch with the highest success rate coincided with a period of a relatively shorter Earth–Moon distance, suggesting a potential but still speculative link to microclimatic conditions.

Perigee conditions produce stronger gravitational pull and higher tidal amplitudes (Bai et al. 2024). These tides may enhance subsurface moisture infiltration, buffering sand temperatures from diurnal heat peaks, a pattern consistent with the stable temperature profile in the highest‐performing clutch. During apogee, weaker tidal influences may increase the likelihood of nest desiccation (Flores‐Aguirre et al. 2023).

Prior research also shows that lunar phases and spring tides influence nest placement and accessibility (Rosenbaum et al. 2023; Srikanthan et al. 2024). Shorter overland travel during spring tides may reduce predation risk and thermal exposure for emerging hatchlings (Martins et al. 2021; Kosaka et al. 2023). These mechanisms remain untested in this study but are consistent with emerging literature suggesting that astronomical variables could indirectly modulate nest environments.

Understanding these mechanisms requires integrating datasets on tides, moisture, and ephemeris. The speculation here serves as a hypothesis‐generating observation consistent with growing attention to lunar–geophysical interactions in sea turtle ecology.

### 3.5 Ecological and Conservation Implications

The descriptive patterns observed in this study reinforce that maintaining thermally optimal, humid, and stable incubation environments supports higher reproductive success. This aligns with findings on olive ridley embryogenesis (Morales‐Mérida et al. 2022). Conversely, the microclimatic instability observed in the less successful clutches mirrors documented risks related to gas exchange, desiccation, and embryonic stress (Gallego et al. 2020; Bomfim et al. 2021).

The potential influence of lunar–tidal conditions offers additional avenues for conservation planning. Aligning nest relocation, shading, supplemental watering, or predator protection with perigee‐associated high tides may enhance nest microclimate stability (Min et al. 2021; Farhat et al. 2022). As climate change increases air and sand temperatures and alters precipitation patterns, such nuanced strategies will become increasingly crucial for safeguarding reproductive output (Laloë and Hays 2023). Integrating microclimate monitoring with astronomical and tidal context may improve predictive capacity for hatchery management and long‐term population viability.

### 3.6 Limitations and Future Research

Interpretation of these findings must consider several limitations. Only three clutches were monitored within a single nesting season, limiting the ability to capture interannual variability in environmental conditions or assess year‐to‐year consistency (Tomillo et al. 2020; Patrício et al. 2021). Environmental parameters were obtained from ERA5 reanalysis rather than in‐nest dataloggers, reducing the resolution of thermal and moisture gradients (Matthews et al. 2021; Fadli et al. 2023). The relatively narrow humidity range restricts the ability to evaluate moisture‐related thresholds (Bomfim et al. 2021; Trewartha et al. 2024). Potential links between lunar cues and microclimate remain inferential without direct measurements of tidal infiltration, porewater content, or geomagnetic variation (Gammon et al. 2023; Bai et al. 2024). Additionally, the study focused only on hatching success, without evaluating sex ratios, hatchling fitness, or emergence success (Lockley and Eizaguirre 2021; Morales‐Mérida et al. 2021).

Future research should adopt multi‐season and multi‐site approaches to capture broader environmental variability. High‐resolution microclimate monitoring using in‐nest sensors would improve understanding of thermal and moisture conditions (Ceolotto et al. 2025; Zhang et al. 2025). Integrating tidal height, porewater movement, and geomagnetic measurements would help test hypotheses about lunar–geophysical influences (Shimada et al. 2021; Yeakub et al. 2023). Experimental manipulations, such as shading, controlled irrigation, or varying nest depth, could clarify causal mechanisms. Including additional metrics such as emergence success, sex ratios, and post‐emergence performance would enable a more holistic evaluation of reproductive ecology under changing environmental conditions (Matthews et al. 2021; Fuentes et al. 2023).

## 4 Conclusion

This pilot study provides preliminary evidence that microclimatic conditions, notably cooler, more thermally stable environments, are associated with higher hatching success in *Lepidochelys olivacea* nests at Batu Hiu Beach. The lunar‐tidal context may indirectly contribute to incubation suitability. Although the small sample size limits broad generalization, the consistent patterns observed across the three clutches align with widely reported thermal and moisture requirements for olive ridley embryonic development and indicate that fine‐scale environmental variability can meaningfully influence reproductive outcomes. These findings highlight the value of continued multi‐season monitoring, in‐nest microclimate measurements, and integrated assessments of terrestrial and astronomical factors to understand better and manage the conditions that support successful incubation in this population.

## Author Contributions

**Titin Herawati:** conceptualization (lead), funding acquisition (lead), methodology (lead), supervision (lead), validation (lead), writing – original draft (lead). **Ismail Maqbul:** formal analysis (lead), visualization (lead), writing – review and editing (lead). **Indriyani Rahayu:** data curation (lead), investigation (supporting), writing – original draft (supporting). **Arifikriyana Saefuramdhan:** data curation (supporting), writing – original draft (supporting).

## Funding

This research was supported and funded by Universitas Padjadjaran, Bandung, Indonesia. The Universitas Padjadjaran Research Grant contract number is 2200/UN6.M/PT.01.03/2025.

## Ethics Statement

This study was approved by the Research Ethics Committee of Universitas Padjadjaran, Indonesia (Approval No. 689/UN6.KEP/EC/2025).

## Conflicts of Interest

The authors declare no conflicts of interest.

## Data Availability

The dataset supporting the results of this study has been deposited in Mendeley Data and is publicly available: Maqbul, I. (2025). *Hatching Success Rate of Olive Ridley Sea Turtles*. Mendeley Data, V1. https://doi.org/10.17632/v23wn4795j.1.
